# The Use of Clear Aligners in Multi-Segmental Maxillary Surgery: A Case–Control Study in Cleft Lip and Palate and Skeletal Class III Patients

**DOI:** 10.3390/jcm13051329

**Published:** 2024-02-26

**Authors:** Maria Costanza Meazzini, Leonardo Paolo Demonte, Noah Cohen, Valeria Marinella Augusta Battista, Dimitri Rabbiosi, Luca Autelitano

**Affiliations:** 1 Regional Center of Cleft Lip and Palate, Department of Maxillo Facial Surgery, Santi Paolo and Carlo Hospital, Via di Rudinì 8, 20142 Milan, Italy; cmeazzini@yahoo.it (M.C.M.); cohen.orthodontic@gmail.com (N.C.); vma.battista@gmail.com (V.M.A.B.); dimitri.rabbiosi@uninsubria.it (D.R.); luca.aute@gmail.com (L.A.); 2 Department of Orthodontics, University Vita-Salute, San Raffaele Hospital, 20132 Milano, Italy; 3 Università degli Studi dell’Insubria, Circolo and Fondazione Macchi Hospital, 21100 Varese, Italy

**Keywords:** invisalign, skeletal class III, craniofacial, orthognathic surgery, segmental Le Fort I osteotomy, occlusogram

## Abstract

**Background**: Maxillary hypoplasia and mandibular asymmetry may be corrected with orthognathic surgery after growth completion. For most stable results, some cases may require segmental Le Fort I osteotomies. Unfortunately, Invisalign’s software (6.0 version) still has some inherent limitations in predicting outcomes for complex surgeries. This study explores the potential of aligners, particularly in multiple-piece maxillary osteotomies in both cleft and non-cleft patients. **Method**: Thirteen patients who underwent pre-surgical treatment with Invisalign were retrospectively matched in terms of diagnosis, surgical procedure, and orthodontic complexity with thirteen patients treated using fixed appliances. Virtual curves following the lower arch were employed to guide the correct pre-surgical positions of the upper teeth with a simple superimposition technique. The amount of impressions required in both groups to achieve satisfactory pre-surgical alignment of the segmented arches was compared. **Results**: one or no refinement phases were needed in the Invisalign group to reach an acceptable pre-surgical occlusion, while the amount of pre-surgical impressions needed to reach adequate coordination with fixed appliance treatment was slightly higher (*p* > 0.05). **Conclusions**: it appears that clear aligner could serve as an effective treatment for individuals necessitating segmental Le Fort I osteotomies when aided by the suggested simple superimposition approach.

## 1. Introduction

Maxillary hypoplasia is a common problem in dysmorphic patients, particularly in patients affected by cleft lip and palate (CLP). It involves the underdevelopment or deficiency of the upper jaw (maxilla). This condition holds significant implications for dental occlusion, speech, and overall function. Furthermore, it profoundly affects facial aesthetics, resulting in a sunken midfacial region, a retruded upper jaw, and insufficient support for the nose and the lips.

The treatment of maxillary hypoplasia in class III patients requires a multidisciplinary approach. Orthognathic surgery often serves as a recommended intervention to advance the maxilla, thereby improving facial balance, in adult patients. It involves careful evaluation and planning by a team of specialists, including oral and maxillofacial surgeons, orthodontists, and speech therapists, aiming for optimal outcomes in function and aesthetics.

In CLP patients, in particular, maxillary hypoplasia may affect all planes of space, with or without mandibular asymmetry. While primary surgery significantly influences maxillary growth, studies indicate that hypoplasia may be present even in unoperated individuals [1]. Many CLP patients present mandibular hypoplasia associated with maxillary hypoplasia (bi-retrusion), reducing the need for mandibular set-back. However, CLP patients exhibit more mandibular asymmetry compared to non-cleft individuals [2]. Orthodontic–surgical treatment is the only viable solution when patients express concern about this asymmetry, although, for the majority of cases, maxillary osteotomy alone may suffice to achieve both aesthetic and functional satisfaction. Le Fort I osteotomy is commonly used for the surgical correction of maxillary skeletal deformities. It was first described by Wassmund in 1921 [3,4]. Patients with skeletal discrepancies need maxillary surgical correction at the completion of growth in all three planes of space in order to achieve the best results [5,6]. When the discrepancy involves either a vertical or a transversal component, the maxilla may typically be divided into two or three segments [7]. In patients with CLP, given the frequent agenesis of the permanent lateral incisor [8], the surgical advancement of the lesser segment is often performed during maxillary osteotomy [9,10].

Orthodontic pre-surgical treatment is often needed to achieve a sufficient coordination between the arches pre-surgically and obtain a more stable post-surgical result by removing all dental compensations. Traditional fixed appliances facilitate the attainment of the ideal pre-surgical occlusion; furthermore, conventional appliances can also be used for intermaxillary fixation and the stabilization of the dental arches after surgery. Dental intercuspation is facilitated and accelerated by the post-surgical RAP phaenomenon [11]. Nevertheless, fixed appliance treatment is burdened by heavy aesthetic shortcomings, which often make it less acceptable to adult patients to face the whole process of orthodontic preparation and post-surgical finishing. Clear aligners seem to be more accepted in terms of aesthetic self-perception, social interaction, and self-consciousness [12,13] but are often considered inadequate for complex orthodontic pre-operative preparation.

Another potential limitation is speech. Speech competence may be negatively influenced by orthognathic surgery, particularly in individuals with a cleft lip and palate who already present borderline speech [14]. Le Fort I advancement may induce an improper closure between the nasal and oral cavities during speech. Changes in the position of the upper jaw can not only affect articulation, but also resonance and overall speech clarity.

Moreover, cleft patients often undergo extensive speech therapy to develop compensatory strategies for effective communication. Orthognathic surgery can disrupt these learned patterns, requiring additional therapy post-operatively to regain or refine speech skills. Segmental Le Fort I osteotomies require a complex orthodontic preparation [15]. Preparation with multibracket orthodontic treatment is the standard procedure. In contrast, orthodontic preparation with clear aligners is not generally used in segmental orthognathic surgery, since most aligner software, ClinCheck (6.0 version) included (Align Technology, Inc., San Josè, CA, USA), do not facilitate prediction when this type of maxillary surgery is chosen, or in cases of asymmetrical movements of the maxilla and the mandible.

The orthodontic pre-surgical treatment consists of removing all dental compensations in all planes of space. All orthodontic movements that tend to relapse should be avoided. In two-piece Le Fort I preparation, transverse expansion should be avoided. In cleft patients, in particular, the lesser segment may be advanced during the osteotomy so that the canine is positioned in the lateral space, left empty by the frequent agenesis of the lateral incisor. In three-piece Le Fort I, transverse expansion as well as anterior tooth extrusion should be avoided, given that those movements will be reached with surgery to ensure better stability. Orthodontic preparation in UCLP patients undergoing the advancement of the lesser segment to close the space of the missing lateral incisor or to improve the intercuspation after the maxillary two-piece advancement involves the palatal movement of the canine crown and the reshaping of the ipsilateral arch so that the first premolar may fit in the canine position, between the lower canine and lower first premolar, after surgery. Canine roots should be tipped distally to allow for safe alveolar surgery when the space of the lateral incisor is already partially or totally closed [16]. When a three-piece segmentation is planned, the roots of the teeth adjacent to the osteotomy sites should be divergent, again ensuring safer surgery. Some patients will need more than one impression during the pre-operative phase in order to achieve adequately coordinated arches. When segmental maxillary surgery is planned, casts need to be cut in the osteotomy site to simulate the surgical procedure.

The aim of this study was to evaluate the results of Invisalign treatment during the preoperative preparation of multi-segment Le Fort I osteotomy, in cleft and non-cleft individuals, aided with a superimposition technique which allows us to draw the correct segmental arch shapes, in comparison with the conventional fixed appliance treatment. Segmental Le Fort I osteotomy often requires an orthodontic preparation phase to adequately facilitate the coordination of the maxillary arch segments to the mandibular arch.

## 2. Patients and Methods

This case-series study was approved by the internal review board of our institution, and it follows the principles of the declaration of Helsinki. Informed consent was obtained from all the participants included in this study (Hospital Internal Ethical Committee HSPC/0022678/2019).

All patients were treated orthodontically by the same orthodontist and subjected to a maxillary segmental osteotomy, with or without mandibular surgery, by the same surgeon.

Since the purpose of this study was not to compare the whole treatment but only to evaluate the adequacy of Invisalign aligners in pre-surgical treatment, the groups were selected in terms of occlusion and orthodontic needs in preparation for surgery. Aetiology of the malocclusion or stability of the surgical result were not considered in this study; therefore, cleft and non-cleft patients were included. All patients were evaluated for speech competence and considered not at risk for the surgical advancement of the maxilla.

### 2.1. Study Sample

**Figure 1:**
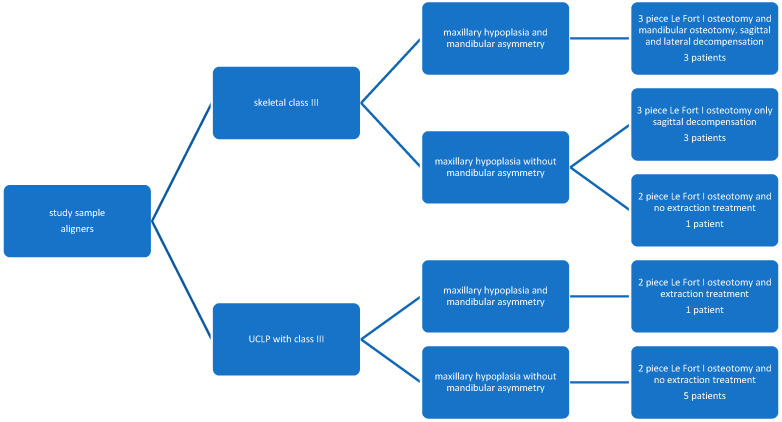
Flowchart depicting the study sample selection showing the different subtypes included, which allowed to retrospectively select a matched control sample.

**Figure 2:**
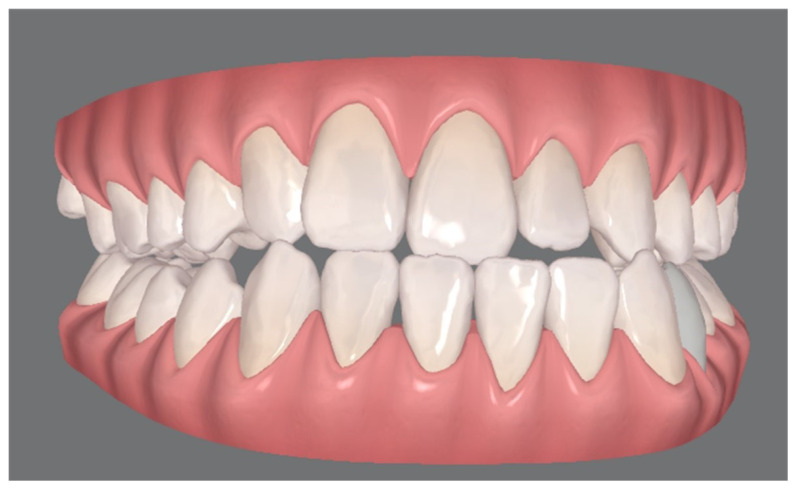
Unilateral cleft lip and palate (UCLP) patient occlusion with class III maxillary and mandibular asymmetry: virtual file of the arches elaborated with ClinCheck^®^ Software.

Thirteen patients treated with Invisalign aligners in preparation for segmental maxillary surgery were included in the study sample. The case-series included seven patients with severe transversal and vertical maxillary hypoplasia with no associated craniofacial anomalies and six patients with unilateral cleft lip and palate (UCLP) aged 19 to 27 (average 22.2 ± 2.9) (Figure 1). All consecutive patients undergoing orthodontic treatment with aligners in preparation of segmental maxillary osteotomies were included in this study, those undergoing one piece le Fort osteotomy were excluded. Regarding maxillary segmentation, surgery six patients in the study sample underwent a 3-piece Le Fort I osteotomy, while seven underwent a 2-piece LeFort 1 osteotomy. One of the UCLP patients and three of the non-cleft patients presented also mandibular asymmetry. These patients also needed an orthodontic pre-surgical lateral decompensation to prepare for the mandibular osteotomy. All patients of the sample underwent sagittal orthodontic decompensation. One patient with UCLP and a non-cleft patient followed an extraction treatment. The UCLP patient with mandibular asymmetry underwent an extraction treatment to centre the mandibular midline, avoid the mandibular osteotomy (Figure 2).

### 2.2. Control Sample

Thirteen patients treated with fixed appliances by the same orthodontist, in preparation for multisegmented maxillary surgery, were retrospectively collected from our centre’s archives and matched for craniofacial anomaly and for the type of maxillary segmentation required (2-piece or 3-piece Le Fort I) with the study sample. We were unable to find in our archives a sample that could be adequately matched for age and sex.

An extremely simplified attempt was made to match patients for complexity of pre-surgical preparation—non-extraction or extraction treatment plan, lateral decompensation (needed/not needed), or sagittal decompensation (needed/not needed)—to prepare for the correction of mandibular asymmetries. Although the matching was only made by large and simple categories, it was carried out by the same orthodontist to compare treatments which might at least be more similar in terms of complexity.

#### Pre-Surgical Orthodontic Treatment

1. Conventional fixed appliances technique

Orthodontic wires were changed periodically and bent, if needed, until the orthodontist determined that the occlusions were potentially ready for surgery. Then, impressions were taken, and casts obtained. The coordination of the segmented arches was assessed by manipulating the casts, which were cut along the osteotomy sites. Preparation for surgery was confirmed when a “good stability” was observed in the segmented pre-surgical models, determined through a qualitative assessment of the contacts made by both the orthodontist and the surgeon, together. Once the preparation was approved, surgical hooks were positioned, and the patient underwent surgery. For patients not yet achieving sufficient coordination, necessary orthodontic adjustments were made, and additional impressions were taken until deemed satisfactory.

2. Clear aligner technique

**Figure 3:**
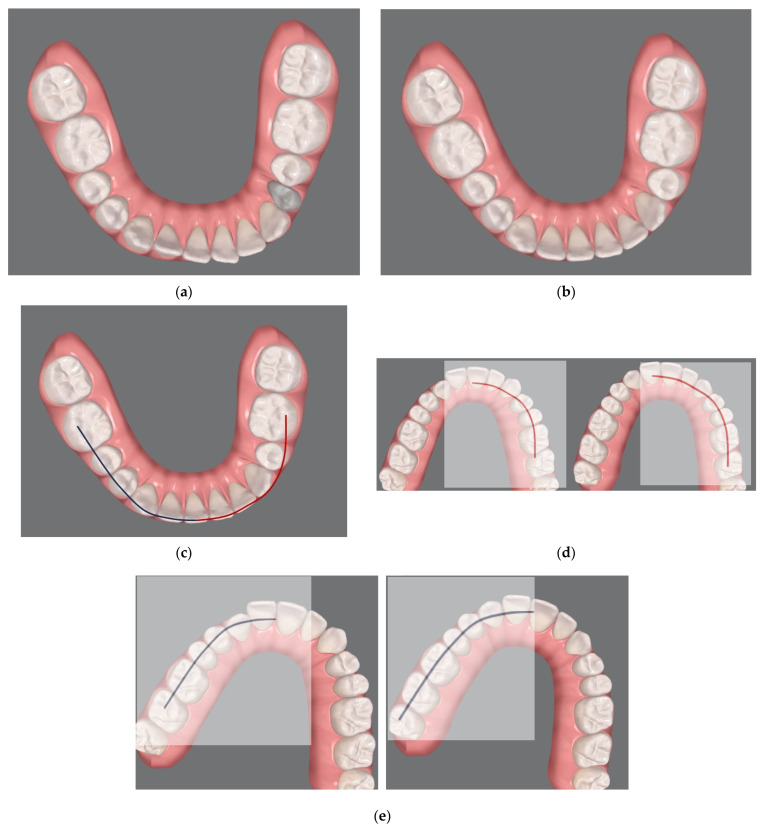
Method developed to coordinate the segments of the maxillary arch to the mandibular arch: (1st step; (**a**,**b**)) initial position of the mandibular teeth and alignment of the mandibular teeth to the correct final position, eliminating compensations; (2nd step; (**c**)) drawing of virtual curves following the final aligned image of the lower arch; (3rd step; (**d**,**e**)) superimposition of the curves on the maxillary arch segments in order to guide the corresponding dental movements to be carried out during the ClinCheck.

Virtual scanning was performed for each patient and a personal virtual file of the arches was elaborated with ClinCheck^®^ Software (Align Technology, Inc., San Josè, CA, USA) (Figure 2). ClinCheck^®^ Software facilitates the manipulation of the location of each tooth independently. Since this software does not allow for the simulation of maxillary segmental osteotomies, to coordinate the segments of the maxillary arch to the mandibular arch, a simple 3-step method, similar to an occlusogram [17], that includes the following actions was developed:

1st step: Align the mandibular teeth in the correct final position, eliminating compensations where needed (Figure 3a).

2nd step: Connect the cuspids of the lower arch in order to obtain the guide curves (Figure 3b).

**Figure 4:**
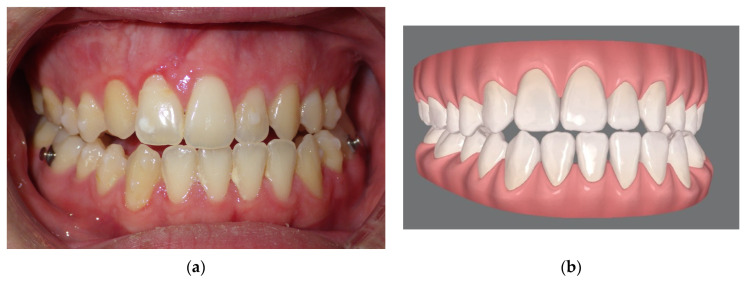
(**a**) Final occlusion obtained by clear aligner pre-surgical preparation. Note that the patient underwent an extraction of the left lower first premolar in order to avoid the mandibular osteotomy and allow for orthodontic decompensation; (**b**) virtual simulation of the final occlusion elaborated with clin check software.

3rd step: Superimpose these curves on the upper arch segments to determine the corresponding final dental positions to be achieved during the Clin Check (Figure 3c,d), obtaining the pre-surgical occlusion (Figure 4).

Both the drawing and the superimpositions of the curves were performed virtually. This superimposition was allowed by the use of an “always-on-top” software (OnTopReplica^®^, 3.5.1 version, 2021 GitHub, Inc., San Francisco, CA, USA). Nevertheless, our protocol is versatile and can be pursued with any other always-on-top software that allows the superimposition of any image on the computer screen. Even acetate paper on the screen could eventually succeed.

At the end of the orthodontic preparation, the criteria used for each patient, by the same orthodontist and the same surgeon, to assess the success of the pre-surgical preparation were the absence of occlusal interferences between the segments and acceptable occlusal stability once positioned in the post-surgical occlusion. Furthermore, to avoid periodontal risks, a radiological assessment of the interradicular space was needed prior to surgery.

Only qualitative criteria were considered to assess the occlusal pre-surgical stability. No quantitative calculation of contact points was carried out. We measured the number of refinements needed before the surgery to assess the adequacy of the pre-surgical orthodontic treatment with Invisalign and compared it with the number of pre-surgical impressions needed prior to surgery in the fixed appliance control group.

Given that the two groups of patients were matched for the complexity of the pre-surgical orthodontic preparation, and not only for the type of surgery and malocclusion, the number of appointments and total treatment time were also compared as additional information to determine whether decompensation with aligners, while more aesthetic, might excessively extended treatment time. A Student *t*-test, setting the *p* value at 0.05, was carried out to compare differences.

## 3. Summary of the Surgical Procedure

**Figure 5:**
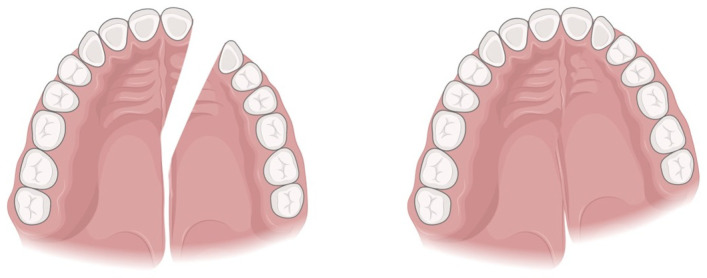
Illustration of a 2-segment osteotomy with advancement of the lesser segment, used in the patient showed as example. Created with BioRender.com by the authors (L.D.).

The osteotomy of the maxilla was usually conduced in three segments. The two-segment osteotomies were placed in the midline in those patients who only required posterior expansion. When the patient was cleft and the osteotomy was planned to close the lateral incisor space by preparing and advancing the lesser segment and positioning the canine in the lateral space, the osteotomy was performed between the central incisor and the canine (Figure 5). It is required to have adequate space for osteotomy, and the roots between the teeth next to the osteotomy should be slightly divergent. All patients of the two groups were operated on by the same surgeon.

One month before surgery, patients of the sample group were bonded to allow for a rigid fixation during and immediately after surgery. Intermaxillary fixation was executed with a final splint setting the position of the maxillary segments. Rigid wire fixation was carried out for one week followed by inter-maxillary elastics for one month and only guidance elastics for the subsequent two months. Three months after surgery, patients of the sample group were debonded, impressions were taken, and they continued with aligners for post-surgical orthodontic finishing.

## 4. Results

Of all Invisalign patients, only four needed one refinement phase after the first set of aligners in order to be considered ready for surgery (average impressions needed was 1.4 ± 0.4; average refinement needed was 0.4 ± 0.4). For all other patients, the surgeon and orthodontists considered the occlusion ready at the end of the first phase. In the fixed appliance sample, we registered an average of 1.8 ± 0.6 impressions (range: one–three) taken during pre-surgical treatment to obtain ideal arch coordination (*p* > 0.05 n.s.).

**Figure 6:**
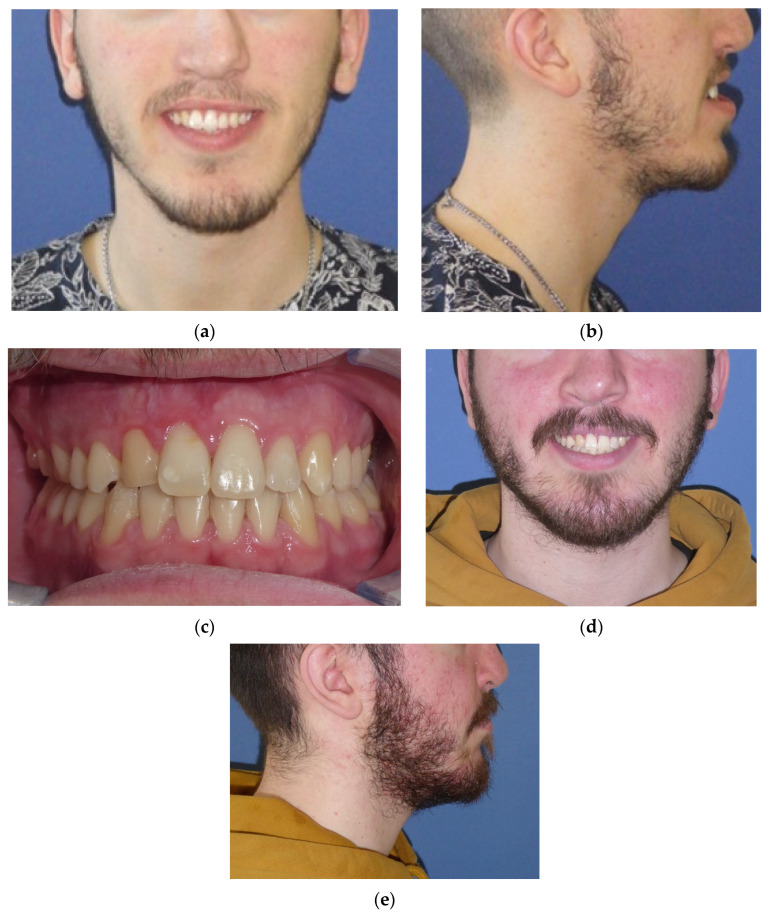
The same UCLP patient: (**a**,**b**) pre-surgical extraoral photographs; (**c**–**e**) intraoral frontal and extraoral post-surgery and orthodontic refinement.

The pre-surgical orthodontic treatment time was similar for the two techniques. The fixed appliance patients were treated for an average of 16.3 ± 4 months (range: 9–22 months) (*p* > 0.05 n.s.), with an average of 10.2 ± 1.6 appointments (*p* < 0.05). The Invisalign patients required an average of 18.4 ± 3 months (range: 11–23) pre-surgically, with an average of 5.1 ± 0.8 appointments. The figures refer to a UCLP patient of the sample group treated with a two-piece Le Fort I osteotomy with a mandibular osteotomy (Figure 6).

**Figure 7:**
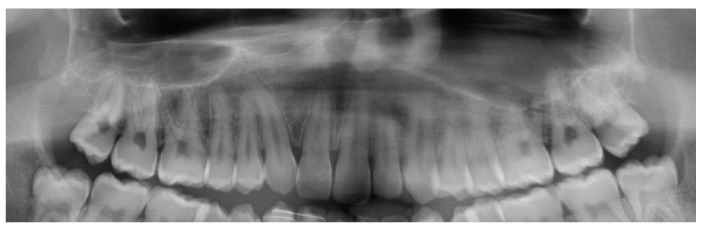
The same UCLP patient’s pre-surgical panoramic showing divergent roots of the central incisor and canine.

In all patients, pre-surgical records demonstrated adequate positions of the roots at the cutting sites, which allowed the surgical procedure (Figure 7).

## 5. Discussion

If a surgical patient asks to be treated with clear aligners, a standard virtual simulation may be performed only for symmetrical maxillary or mandibular sagittal movements using the ClinCheck software. Invisalign software does not allow for virtual cutting of the arches and it is only possible to simulate a single-segment Le Fort I osteotomy without any rotation of the maxilla.

This retrospective case-series study suggests that the Invisalign software may be used in patients who need segmental Le Fort I osteotomy, or asymmetric osteotomies, applying the simple method recommended. The use of this additional three-step work up has shown that it is possible to achieve a satisfactory coordination with only one pre-surgical ClinCheck or one additional refinement phase at the most.

There are not many articles discussing the treatment with clear aligners as orthodontic preparation before orthognathic surgery. A study by Kankam [18] intended to assess post-operative outcomes in patients treated with Invisalign and conventional fixed appliances; however, they only reported on post-operative oedema and did not investigate other outcomes, such as treatment time, patient satisfaction, or arch coordination.

Though the accuracy of the Invisalign technique for dental movements is reported to be only 60% to 80% [19,20] compared to fixed appliances, it appears acceptable to plan and achieve a pre-surgical preparation that allows for a sufficient occlusal pre-surgical stability [21,22]. The accuracy in terms of occlusion variables in a pre-surgical preparation is very different from the accuracy required for final orthodontic detailing. At the completion of non-surgical orthodontics, multiple occlusal contacts are expected and required for maximum functional results [23]. On the other hand, the definition of a stable pre-surgical occlusion is subjectively judged by the orthodontic–surgical team, seeking a sufficiently stable intercuspation of each segment, relative to the mandibular arch. In our cases, the occlusal evaluations were made by the same surgeon and orthodontist, both for the sample and control groups, allowing for a more reliable comparison, although the fact that the stability was not assessed quantitatively is an obvious major limitation in our study.

The similarity in treatment duration suggests that the matching for severity was reasonably accurate. The matching of samples was not entirely precise, and patient compliance introduces an additional variable in the Invisalign group that could potentially skew treatment timing. We initially examined the treatment time, only out of concern that employing clear aligners for complex three-dimensional preparation would increase the treatment time significantly and thus prove overly burdensome in terms of care requirements. The results of this small study seem to point out that the difference in treatment time is not meaningful. Conversely, the discrepancy in appointment frequencies may suggest that Invisalign might be a favourable choice for patients who must travel considerable distances to access the centre.

Before surgery, two impressions were consistently taken: the first after the initial set of aligners, to assess occlusion, and the second, three weeks post passive orthodontic bonding, to ensure no unintended dental shifts had occurred during the fixed appliance time and to fabricate the surgical splint. This is an evident drawback of this method. It is possible to bypass this disadvantage by carrying out the post-surgery fixation only with bone-anchored screws, but many centres avoid the use of surgical screws for a number of reasons. Orthognathic surgery screws are very bulky and often not well accepted by the patients. They do not allow for early post-surgical movements to improve intercuspation with triangular elastics which are facilitated by the RAP (regional acceleratory phaenomenon) induced by the surgery itself [11]. This is a period of rapid metabolic activity within the bone and periodontal tissues which facilitate and accelerate efficient orthodontic tooth movement. Orthognathic surgery screws are also particularly expensive for centres where everything is paid for by the national health service.

In bimaxillary osteotomies with multi-segment Le Fort I, fixed appliances are preferred by our surgeon in the fixation of the skeletal segments and bases, both during surgery and through the post-operative healing period. At our centre, although they represent an alternative to dental post-surgical fixation, no screws for skeletal anchorage are provided by the hospital.

In this study, cleft and non-cleft orthognathic surgery patients were included in the series. Although surgery might be more complex and the stability less predictable in clefts [24], the orthodontic steps for multi-segment maxillary preparation are identical.

This approach does not require specific computer skills or a learning curve. Having expertise in surgical cases and the capability to execute a basic occlusogram for orthodontists [17] suffices. Additionally, one can perform these same procedures using acetate paper placed over a computer screen. While more advanced software will most certainly replace this method in the future, it will still operate on the same underlying principles.

Other limitations of our study are the lack of both the reproducibility of the method and comparisons between the virtually planned pre-surgical occlusion and the actual one. The plaster casts used to plan the surgery were mostly not available at the time of this retrospective assessment and, therefore, no quantitative retrospective assessment of occlusal contacts was possible.

Therefore, to summarize, the limitations of this study are its retrospective nature, which has an inherent selection bias; the fact that the assessment of readiness for surgery was a qualitative team assessment, which, although always carried out by the same operators for all patients in this study, nevertheless, did not allow for any reliability testing; and the superimposition method itself, although deriving from a very well standardized pre-treatment visualization approach [17], is simply the suggestion of an easy stepwise method that may be used by clinicians to improve the quality of pre-surgical preparation at this time, but it shall certainly be substituted by a more technologically advanced software in the future.

## 6. Conclusions

Patients with skeletal disharmonies and complex craniofacial anomalies, requiring multisegmented Le Fort I osteotomies or asymmetrical movements, usually need to undergo a psychologically burdensome phase of pre-surgical orthodontic decompensation and segmental preparation with multibracket orthodontics. While standard multibracket pre-surgical treatment remains the most common choice, using the simple superimposition method suggested in this study during the digital simulation for pre-surgical movements, it appears that clear aligner therapy could be a suitable alternative treatment option, with the advantage of a more aesthetic appliance combined with the possibility of fewer appointments. The method described might serve as a suggestion for the development of future software adjustments in the simulation tools of any orthodontic aligner system.

## Data Availability

The data that support the findings of this study are available on request from the corresponding author (L.D.)
